# Responsible research and innovation training programs: implementation and evaluation of the HEIRRI project

**DOI:** 10.1080/20961790.2021.1970319

**Published:** 2021-11-02

**Authors:** Ružica Tokalić, Ivan Buljan, Niels Mejlgaard, Mar Carrió, Alexander Lang, Gema Revuelta, Ana Marušić, HEIRRI Consortium

**Affiliations:** a Department of Research in Biomedicine and Health, University of Split School of Medicine, Split, Croatia; b Danish Centre for Studies in Research and Research Policy, Department of Political Science, Aarhus University, Aarhus, Denmark; c Health Sciences Education Research Group, Department of Experimental and Health Sciences, Universitat Pompeu Fabra, Barcelona, Spain; d Institut für Höhere Studien – Institute for Advanced Studies (IHS), Vienna, Austria; e Studies Centre on Science, Communication and Society, Department of Experimental and Health Sciences, Universitat Pompeu Fabra, Barcelona, Spain

**Keywords:** Research integrity, research ethics, responsible research and innovation, responsibility, societal inclusion, higher education institutions, RRI teaching

## Abstract

Responsible research and innovation, or RRI, is a concept that aims to bring together society and science for a better future. There are six key elements of RRI: public engagement, gender equality, science education, open access, ethics and governance.

Higher Education Institutions and Responsible Research and Innovation (HEIRRI) project aimed to bring the concept of RRI into the educational system. Using state-of-the-art review of good practices, HEIRRI team developed 10 training programs on RRI for different higher education institution educational levels, including a summer school and a massive open online course (MOOC). We conducted pilot of the trainings and evaluated participants’ experiences. Satisfaction with HEIRRI training programs on responsible research and innovation was high, both for participants and for the trainers, and trainings raised awareness of RRI. Participants’ feedback was used to identify areas that need improvement and provided for recommendations for final versions of the HEIRRI training programs.

In order to equip researchers with skills to recognize and apply RRI values, RRI should be included in their education. HEIRRI training is suitable for a range of different disciplines, including forensic science, and is free to use and adjust for specific contexts (available from: https://rri-tools.eu/heirri-training-programmes).

Supplemental data for this article is available online at https://doi.org/10.1080/20961790.2021.1970319 .

## Key points

Responsible research and innovation (RRI) aims to make research inclusive, responsible, reflexive and to foster integrity of researchers

- HEIRRI project developed a toolbox of engaging activities for introduction of RRI concepts in higher education
- HEIRRI training can be used to introduce RRI at all levels of higher education and members of the public
- HEIRRI training is suitable for a range of different disciplines, including forensic science, and is free to use and adapt

## Introduction

Research integrity is an important building block of responsible conduct of research, which includes a responsibility towards society. Responsible research and innovation, or RRI, is a concept that aims to bring together society and science to contribute to a better future. To achieve this, RRI encourages key stakeholders to be aware and responsive to the needs and values of society, through a value-based ­framework [1].

There are multiple origins of RRI as we know it today, from ‘science and technology studies’, to ‘anticipatory governance’, ‘value sensitive design’, and ethics of development [2]. The origin of the modern RRI concept can be found in technology assessment, a policy-advising activity developed in the USA in the 1970s [3]. The aim of this policy was originally to anticipate and prevent negative consequences of new technologies, and later it tried to shape those technologies according to the needs of society [4]. This societal model developed into the responsible research and innovation concept we know today.

In the Rome Declaration on Responsible Research and Innovation in Europe, RRI is defined as an “on-going process of aligning research and innovation to the values, needs and expectations of society” [5]. Von Schomberg defines it as “a transparent, interactive process by which societal actors and innovators become mutually responsive to each other with a view to the (ethical) acceptability, sustainability and societal desirability of the innovation process” [6]. The European Commission recognized six key elements of RRI: public engagement, gender equality, science education, open access, ethics and governance [7].

RRI thus escapes a uniform definition, and lives as an emerging application of reflexive practices in different fields of research and science. One of its crucial parts and what it promotes is the inclusion of societal actors in decision making, in order to enable shaping of the future according to societies’ needs and wishes. It advocates open access – to data, to work, to implications it delivers, to decisions and to arising ethical dilemmas. It endorses gender equality, ethical development, environmental consciousness and informed citizenship [3].

When addressing the way in which researchers should behave and work, or more often, the ways in which they should not, research ethics (RE), research integrity (RI) and responsible conduct of research (RCR) are the concepts used to explain and direct action. While these concepts are commonly seen as overlapping, their relationship with RRI is not as clear. Ethics is one of the key parts of RRI. Research integrity is oriented inward and focuses on how researchers should behave, while RRI is oriented towards the outside world and society [8]. However, to have integrity as a researcher, one needs to be aware of their societal responsibility, to their colleagues, research participants and society in general [9]. Open access and open science are an overarching theme for RI, RCR and RRI, as they include sharing not only research results and data, but also sharing uncertainties, risks, implications and potential use of research results [10].

Horizon 2020, the largest European Union research program, has recognized the importance of open and participatory science. The Science with and for Society (SwafS) program of the European Commission aimed to support the evolution of science and society with an emphasis on their relationship [11]. One of the SwafS projects, Higher Education Institutions and Responsible Research and Innovation, or HEIRRI [12], aimed to bring the concept of RRI into the educational system, particularly those of higher education institutions. Higher education institutions were defined as educational institutions available after secondary education – universities, academies, colleges, seminaries, and institutes of technology [13]. The HEIRRI team developed a state-of-the-art review and a database [13], consisting of good practices and cases, teaching materials, and results from other RRI projects. One of the key findings of this review was that there are numerous educational resources that encompass RRI key aspects in some way, but not the concept of RRI as a whole. Higher education institutions still struggle with structural changes needed for optimal implementation of RRI education. A supportive environment is important for RRI teaching. Focussed and dedicated RRI teaching activities, across organizational activities and levels of research, teaching, innovation, and societal engagement, can contribute to organizational change [13].

Using these results, the HEIRRI team developed 10 training programs on RRI for different higher education institution educational levels, including a summer school and a massive open online course (MOOC) [14]. The programs are mainly based on problem-based learning (PBL) methodology, led by case-based scenarios, with a strong focus on reflection and dialogue, and supported by videos, scenarios, and card games. Programs for undergraduate studies are designed as modules that can easily be included within existing subjects, such as bioethics or science communication, across a wide range of disciplines, from biomedical and forensic sciences to humanities and social sciences. All the HEIRRI materials are available in open access at the RRI Tools platform [15].

In this study, we evaluated the pilot training experiences, both in HEIRRI consortium institutions and institutions outside of the consortium.

## Methods

### Study design and setting

To evaluate the quality of the HEIRRI pilot programs, we used the quasi experimental design, with post-interventional assessment only. Overview of the programs is presented in Table s1 in the **Supplement**. The RRI programs were held at five HEIRRI project consortium institutions (University of Split, Croatia; Pompeu Fabra University, Spain; University of Bergen, Norway; Aarhus University, Denmark; and Institute for Advanced Studies, Austria). The non-consortium member institutions in the program evaluations were: Institute of Water and Energy Sciences, Pan African University (Algeria); Centre for Studies in Science Policy, Jawaharlal Nehru University (India); Universitat Autònoma de Barcelona (Spain); Christian Albrechts Universität zu Kiel (Germany); Aachen University (Germany); Universitat Jaume I (Spain); Sofia University (Bulgaria); University of Mostar School of Medicine (Bosnia and Herzegovina); Mykolas Romeris University (Lithuania); and Universitat Oberta de Catalunya UOC (Spain). The full list of institutions and pilot trainings is available in Tables s2 and s3 in the Supplement.

**Table 1.** Demographic characteristics of participants (*n* = 555)*.

Characteristic	No. (%)
Age group (in years, *n* = 427)	
15-24	108 (25.3)
25-34	192 (45.0)
35-44	86 (20.1)
45-54	27 (6.3)
55-64	13 (3.0)
≥65	1 (0.2)
Gender (*n* = 441):	
Female	253 (57.3%)
Male	188 (42.7%
Consortium status (*n* = 511):	
Member	232 (45.4%)
Non-member	279 (54.6%)
Previously involved in research activities (*n* = 413)	
Yes	302 (73.1%)
No	111 (26.9%)

*Number of answers to each question is indicated in the brackets.

**Table 2.** Course satisfaction level, attitudes towards responsible research and innovation (RRI) and intention for future use of RRI of male and female participants, consortium and non-consortium members and participants who had been involved in research compared to those who had not (*n* = 555).

Measure*	Characteristic (Md, 95% CI)		*P*†
**Gender**	**Male (*n* = 188)**	**Female (*n =* 253)**	
Course satisfaction (*n* = 79)	72.7 (761.2 to 75.8)	75.8 (74.2 to 78.8)	0.021
RRI Attitudes (*n* = 99)	67.9 (65.5 to 72.6)	67.9 (66.7 to 70.2)	0.543
Intention for future use (*n* = 87)	86.7 (83.3 to 90.0)	90.0 (86.6. to 93.3)	0.029
**Consortium status**	**No (*n* = 279)**	**Yes (*n* = 232)**	
Course satisfaction (*n* = 59)	78.8 (75.6 to 80.3)	74.2 (72.7 to 77.3)	0.005
RRI Attitudes (*n* = 79)	75.0 (72.6 to 78.6)	66.7 (64.3 to 69.1)	**<0.001**
Intention for future use (*n* = 78)	93.3 (93.3 to 96.7)	86.7 (83.3 to 90.0)	**<0.001**
**Involved in research activities?**	**No (*n* = 111)**	**Yes (*n* = 302)**	
Course satisfaction (*n* = 57)	72.8 (68.2 to 75.8)	75.8 (74.2 to 78.8)	0.047
RRI attitudes (*n* = 75)	70.2 (66.7 to 72.6)	65.5 (63.1 to 67.9)	0.051
Intention for future use of RRI (*n* = 65)	90.0 (86.7 to 93.3)	86.7 (80.0 to 93.3)	0.789

Md – median, CI – confidence interval.

*Missing values indicated in brackets. The scores on individual measures range from 0 to 100.

†Mann-Whitney test, Bonferroni correction (0.05/(n of comparisons = 9). *P* significance level < 0.005. Significant *P*-values are indicated in bold.

### Participants

The participants were university students from different educational programs, professionals from academia/research from the consortium members’ or from other institutions, and members of the general public partaking in the HEIRRI museum training activities. Participation in the courses was voluntary and the participants were recruited by consortium members and their network of contacts. The data collection was performed at the end of the course programs, and data were collected using the pen and pencil approach or online software (SurveyMonkey, San Mateo, California, USA).

The implementation of the pilot programs into the education activities at HEI has already been approved for the HEIRRI grant by all consortium members. The overall ethics approval for the surveys was obtained by the University of Split School of Medicine, as the coordinator of the survey (Register Number: 2181-198-03-04-17-0019, Class: 003-08/17-03/0001); other partners confirmed that an ethics approval for social science research was not required according to their national/university regulations. For training activities and surveys outside of the consortium, the participating institutions obtained necessary approvals.

### Programs

The programs we tested were courses developed by the HEIRRI consortium (Table s1 in the **Supplement**). In total, there were ten courses, designed for populations with different levels of expertise in RRI. The courses focused on the implementation of RRI concepts in different disciplines and had various duration and audience. The full description of the courses can be found at the RRI Tools platform [15].

### Outcomes

Pilot training programs were evaluated through surveys that addressed attitudes and perceptions of participants and teachers.

We used the four-level typology of outcomes described by Kirkpatrick and modified by Barr *et al* [16, 17] to develop the questions for the survey:

*Level 1* outcomes refer to learners’ reaction to the intervention, including participants’ views of their learning experience and satisfaction with the program.

*Level 2* outcomes refer to changes in attitudes and knowledge:

*level 2a* outcomes refer to modification of attitudes and/or perceptions regarding responsible conduct of research;

*level 2b* outcomes refer to acquisition of knowledge and/or skills related to responsible conduct of research.

*Level 3* outcomes refer to behavioral change transferred from the learning environment to the workplace prompted by modifications in attitudes or perceptions, or the application of newly acquired knowledge/skills in practice. This level can be further divided as follows:

*level 3a* outcomes related to behavioral intentions; and

*level 3b* outcomes related to actual change in practices.

*Level 4* outcomes refer to organizational changes attributable to the intervention.

Surveys also included a qualitative part, where participants and teachers were asked to describe their experiences and opinions in a free-text format.

#### Development and availability of the instruments

To create an initial set of candidate items, the UNIST team organized a brainstorming session and generated up to 20 Likert-type statements for each of the three levels of measurement (1, 2a and 3a), addressing four RRI dimensions (except for the questions addressing satisfaction with the training program). The statements were either positive or negative. The scale had 7 scoring points, from 1 (“strongly disagree”) to 7 (“strongly agree”), with 4 as a neutral point (“neither agree nor disagree”). Then, at least 14 of the most relevant statements for each of the three instruments were selected through the consultation process with UPF members. The three surveys were then piloted with five independent experts (including two university senior professors and three PhD students) to test their face-validity. Pilot-testing resulted in the wording changes for five of the statements to improve their clarity. The final form of the surveys can be found in the Tables s4 to s6 in the **Supplement**.

**Table 4.** Proportions of commonly addressed topics in open-ended survey question for student pilot participants (*n* = 325).

Topic (total number of answers with topic)	Proportion of topic in answers
Great introduction to RRI* (*n* = 106)	32.6%
Useful (*n* = 84)	25.9%
Good design (*n* = 59)	18.2%
Could relate to work (*n* = 27)	8.3%
New point of view (*n* = 23)	7.1%
Great resources (*n* = 22)	6.8%
Important (*n* = 22)	6.8%
Intention to use (*n* = 21)	6.5%
Motivating (*n* = 19)	5.6%
Multidisciplinary (*n* = 9)	2.8%
Suggestions for improvement:	
More real-world examples (*n* = 47)	14.5%
More time needed (*n* = 36)	11.1%
Clearer task explanation (*n* = 23)	7.1%
More flexibility (*n* = 19)	5.9%
More theoretical resources (*n* = 6)	1.9%
Unclassified/other (*n* = 29)	8.9%

*RRI – responsible research and innovation.

The developed surveys addressed level 1, level 2a, and level 3a from the typology described above. The surveys assessing level 1 (reaction to the training program) and level 2a (attitudes and perceptions) were used to assess the impact of the training programs from the perspective of the teachers and stakeholders (involved in pilot experiences in science museums). Survey questions related to levels 2a and 3a addressed the four dimensions of RRI: diversity and inclusion, anticipation and reflexivity, responsiveness and adaptation, openness and transparency.

##### 1) Level 1 outcomes: Course assessment

This part of the survey had 11 items. Higher scores indicated greater satisfaction with the RRI course. Three items (items No. 1, 8 and 10) were reversely scored. The final score was the sum of all answers with theoretical range from 11 to 77, and Cronbach’s alpha was α = 0.74 (95% confidence interval, CI = 0.71 to 0.77).

##### 2) Level 2a outcomes: Attitudes towards RRI

This part of the survey had 12 items. Higher scores indicated more positive attitudes towards RRI. Five items (items No. 1, 2, 3, 6, 7) were reversely scored. The final score was the sum of all answers with theoretical range from 14 to 98, and Cronbach’s alpha was α = 0.80 (95% CI = 0.78 to 0.83).

##### 3) Level 3a outcomes: behavioural intentions

The component of the survey had 5 items. Higher scores indicated greater intention to use RRI in the future research projects. The final score was the sum of all answers with theoretical range from 5 to 35, and Cronbach’s alpha was α = 0.90 (95% CI = 0.89 to 0.92).

### Statistical methods

Total scores for course satisfaction, RRI attitudes and intention for future use were calculated only for participants who had answers to all survey items. To present the results more clearly, and to compare the differences between different outcomes relevant to program evaluation, the survey scores were transformed to the scale range 0-100, using the following formula: (S-m)/(M-m) ×100; where S is the total score, m is the minimal theoretical total score value and M is maximal theoretical total score value [18]. Due to the non-normality of the distribution of the results, the survey results are presented on the group level and are presented as medians with 95% confidence intervals (CI). The differences between male and female participants, participants from the consortium member’s institutions and participants from institutions outside of the consortium, and participants who were involved in research activities compared to those who were not, were compared using Mann-Whitney test, with Bonferroni correction in order to avoid alpha error. The statistical analysis was performed using MedCalc Statistical Software [19].

We also analyzed the content of the comments related to the course assessment using Linguistic Inquiry and Word Count program, a software for computerized language analysis [20]. LIWC has an established dictionary and counts words related to specific category. Word count refers to the number of words used in the text for specific LIWC categories. In the current case, individual comments were used as the unit of analysis.

All LIWC linguistic variables range from 0-100, indicating the percentage of the words in the text related to specific category, and the interpretation of the LIWC summary variables is the following:

- Analytical thinking is a dimension which is related to formal and scientific thinking, and higher scores indicate objectivity and “cold” writing in the text.
- Clout is a tone related to words written from a position of superiority and power. The comments with high clout indicate confidence.
- Authenticity refers to the honest writing style, with words that indicate vulnerability and higher use of personal pronouns.
- Emotional tone is a dimension where higher scores (over 50.0) are related with the texts with positive emotions.

Comments were analyzed for common topics. Frequency of the topics was calculated and presented as proportions, along with the total number of answers with individual topics. Comments that did not fit within the common identified topics (i.e. whose tags were represented in <2% of the comments), were marked as unclassified, as assigning them individual tags would lead to over-categorization. We also calculated the frequencies for all the words participants used to describe their experience in open-ended questions, and presented those as a word cloud. For topics and word frequency, we used NVivo 12 Plus for Windows [21] (Figure 1).

## Results

In total, 555 people (*n* = 253, 57.3% female, of participants who stated their gender) participated in courses, the majority of whom (*n* = 302, 73.1% of available answers) were involved in research activities (Table 1). Participants rated the overall courses positively, above average (median (Md)=75.7 out of maximum 100 score points, 95% CI = 74.2 to 78.8, *n* = 470), but there were no gender differences in course satisfaction, attitudes towards RRI or intention for future use. Participants reported positive attitudes towards RRI in general (Md = 71.4, 95% CI = 69.1-73.8, *n* = 449) and intention for future use (Md = 90.0, 95% CI = 90.0 to 93.3, *n* = 446). Non-consortium institution members had more positive attitudes towards RRI and higher intention for future use, compared to consortium members (Table 2). Previous research activities did not play a role in course satisfaction, attitudes towards RRI or intention for future use (Table 2). Results per individual survey item are presented in Tables s7 to s9 in the Supplement.

**Figure:**
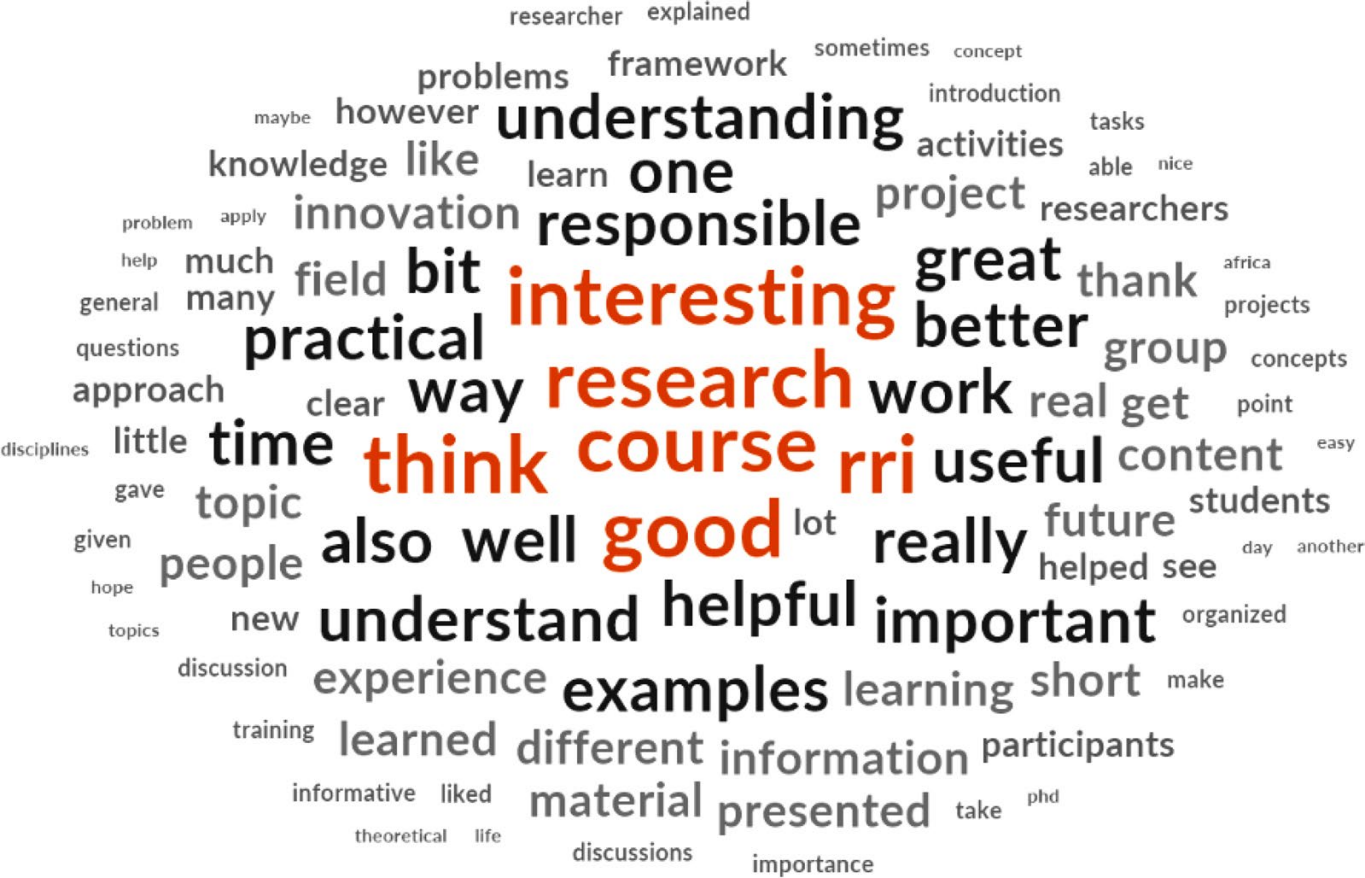
Frequency of words used in participants’ comments generated by NVivo 12 Plus for Windows.

### Comments analysis

Only comments in English were analyzed for linguistic characteristics (total *n* = 277). Overall, participants’ comments were brief, positive (high scores on “Tone” variable) and informally written (low scores on “Clout” variable). There was no difference in linguistic characteristics of the feedback related to participants’ gender, consortium status or previous research activities (Table 3).

**Table 3.** Comparison of LIWC scores of course feedback comments in relation to gender, consortium status and previous involvement in research activities*.

Measure	Characteristic (Md, 95% CI)		*P*†
Gender	Male (*n* = 107)	Female (*n* = 145)	
Word count	16 (10 to 23)	20 (16-22)	0.171
Analytical tone	80.8 (54.3 to 88.9)	72.0 (60.8 to 78.4)	0.228
Clout	50.0 (50.0 to 50.0)	50.0 (50.0 to 59.9)	0.018
Authenticity	13.5 (5.8 to 26.0)	17.5 (13.2 to 26.6)	0.771
Emotional tone	99.0 (93.6 to 99.0)	99.0 (97.3 to 99.0)	0.563
Consortium status	No (*n* = 151)	Yes (*n* = 117)	
Word count	18 (14 to 21)	20 (15 to 24)	0.328
Analytical tone	74.9 (60.8 to 82.0)	77.3 (67.1 to 83.0)	0.655
Clout	50.0 (50.0 to 50.0)	50.0 (50.0 to 50.0)	0.778
Authenticity	17.5 (11.0 to 26.3)	17.5 (9.6 to 23.1)	0.902
Emotional tone	99.0 (96.8 to 99.0)	99.0 (98.0 to 99.0)	0.641
Involved in research activities?	No (*n* = 65)	Yes (*n* = 184)	
Word count	19 (13 to 22)	18 (15 to 21)	0.582
Analytical tone	74.9 (58.1 to 82.0)	76.5 (66.8 to 82.0)	0.890
Clout	50.0 (50.0 to 50.0)	50.0 (50.0 to 50.0)	0.357
Authenticity	17.5 (10.1 to 29.6)	16.1 (8.9 to 23.5)	0.349
Emotional tone	99.0 (96.8 to 99.0)	99.0 (97.6 to 99.0)	0.597

LIWC – Linguistic Inquiry and Word Count software, Md – median, CI – confidence interval.

*The range for linguistic characteristics (Analytical tone, Clout, Authenticity and Emotional tone) is from 0-100.

†Mann-Whitney test for independent samples. Bonferroni correction (0.05/(n of comparisons = 9). *P* significance level < 0.005.

Participants found the design of the courses to be appropriate. They also stated they could relate course content to their work, and that they appreciated new and different points of view that the courses provided them. Participants also used comments to suggest possible improvements. Common suggestions for improvements were as follows: need for more real-world examples, as well as examples from different fields of research; lack of time for more detailed discussion of the materials; and clearer task explanations and more flexibility in program scheduling and execution. Commonly addressed topics in the open-ended answers are presented in Table 4. A sample of open-ended answers is available in Table s10 in the Supplement.

**Table 10.** Proportions of commonly addressed topics in ope- ended survey question for trainers (*n* = 35).

Topic (total number of answers with topic)	Proportion of topic in answers
Motivating, interactive (*n* = 14)	40.0%
Good design (*n* = 9)	25.7%
Great introduction to RRI (*n* = 5)	14.3%
Intention to use (*n* = 4)	11.4%
Great resources (*n* = 3)	8.6%
Suggested improvements:	
More materials and examples (*n* = 11)	31.4%
More time needed (*n* = 9)	25.7%
More instructions (*n* = 4)	11.4%
Should be shorter (*n* = 3)	8.6%
Unclassified/other (*n* = 3)	8.6%

****

### Trainers

Most trainers were in the 35-44 age group, female, and from non-consortium institutions (Table 5). Trainers expressed high levels of satisfaction with the course, resources and participant interactions (Table 6), with above average scores (standardized score Md = 77.1, 95% CI = 70.8 to 79.2).

**Table 5.** Demographic characteristics of trainers (*n* = 48)*.

Characteristic	No. (%)
Age group (in years, *n* = 48)	
25-34	6 (12.5)
35-44	20 (41.7)
45-54	10 (20.8)
55-64	11 (22.9)
65 and older	1 (2.1)
Gender (*n* = 48):	
Female	26 (54.2)
Male	22 (45.8)
Consortium status (*n* = 44):	
Member	14 (31.8)
Non-member	30 (68.2)
Previously involved in research activities (*n* = 46)	46 (100.0)

*Number of answers to each question is indicated in the brackets.

**Table 6.** Feedback from trainers.

Statement	n*	Response (Md, 95% CI)†
1. Overall I am very satisfied with the program and training materials.	43	6.0 (6.0 to 6.4)
2. I wish I had had more time to cover all of the training contents.‡	43	5.0 (4.0 to 6.0)
3. Participants were very active during the training.	43	6.0 (6.0 to 7.0)
4. Course resources were appropriate.	43	6.0 (6.0 to 6.0)
5. I had difficulties in finding examples to illustrate particular topics during the training. ‡	41	2.0 (2.0 to 2.0)
6. There was sufficient interaction between the participants during training.	41	6.0 (6.0 to 6.0)
7. It was easy to motivate participants to take part in the training activities.	41	6.0 (6.0 to 6.2)
8. The training was difficult to embed in the discipline I teach.‡	41	2.0 (1.0 to 3.0)

Md – median, CI – confidence interval.

*Number of respondents for each question, as some answers were not provided by the participants.

†Numbers refer to selected scores on Likert answer scale with 7 scoring points, from 1 (“strongly disagree”) to 7 (“strongly agree”), with 4 as a neutral point (“neither agree nor disagree”).

‡Negative statements, reverse scoring was used.

Pilot trainers agreed that the programs were well designed, motivating and interactive. They expressed a need for more real-world examples in the materials, as well as a need for more scheduled time for some of the exercises. Commonly addressed topics in the open-ended answers are presented in Table 7. A sample of open-ended answers is available in Table s11 in the Supplement.

**Table 7.** Proportions of commonly addressed topics in open-ended survey question for pilot trainers (*n* = 35).

Topic (total number of answers with topic)	Proportion of topic in comments
Motivating, interactive (*n* = 14)	40.0%
Good design (*n* = 9)	25.7%
Great introduction to RRI (*n* = 5)	14.3%
Intention to use (*n* = 4)	11.4%
Great resources (*n* = 3)	8.6%
Suggested improvements:	
More materials and examples (*n* = 11)	31.4%
More time needed (*n* = 9)	25.7%
More instructions (*n* = 4)	11.4%
Should be shorter (*n* = 3)	8.6%
Unclassified/other (*n* = 3)	8.6%

### Public participants

Participants reported that museum activities were interesting and engaging, and expressed intentions for learning and participating more in the future (Md = 69.1, 95% CI = 61.9 to 76.2) (Table 10). Their RRI attitudes, measured after activities, were also positive above average (Md = 66.7, 95% CI = 60.0 to 76.7) (Tables 8 and 9).

**Table 8.** Satisfaction of participants with museum ­activities and intentions for future behaviour (*n* = 24).

Statement	n*	Response (Md, 95% CI)†
1. The activity in the museum was interesting.	23	6.0 (6.0 to 6.0)
2. I actively participated in the activity in the museum.	23	6.0 (5.0 to 6.0)
3. I learned a lot about responsible research and innovation (RRI).	23	5.0 (4.0 to 5.0)
4. I am now curious about research that is important and relevant for me.	23	5.0 (5.0 to 6.0)
5. In future, I will learn more about research that is directly relevant and important for me.	23	6.0 (5.0 to 6.0)
6. In future, I would like to participate in planning research that is relevant and important for me.	23	6.0 (5.0 to 6.0)
7. How informed are you about new scientific discoveries and technological developments?	22	5.0 (3.0 to 6.0)

Md – median, CI – confidence interval.

*Number of respondents for each question, as some answers were not provided by the participants.

†Numbers refer to selected scores on Likert answer scale with 7 scoring points, from 1 (“strongly disagree”) to 7 (“strongly agree”), with 4 as a neutral point (“neither agree nor disagree”).

**Table 9.** Attitudes towards RRI of participants in museum activities (*n* = 24).

Statement	n*	Response (Md, 95% CI) †
1. I think the public should be more engaged in research and innovation.	23	6.0 (6.0 to 6.0)
2. I think that the public has little to contribute to the development of research projects.‡	23	4.0 (3.0 to 6.0)
3. I think that the scientists are very responsive to the needs of the society.	23	5.0 (4.0 to 5.0)
4. I am familiar with sources of information where I can follow ongoing research projects that are important for me.	22	5.0 (4.0 to 6.0)
5. Scientists are very transparent in their work.	23	5.0 (4.0 to 5.0)
6. More active science education would help the public to be more engaged in research.	22	6.0 (5.0 to 6.0)
7. The public can help scientists to shape their research to address the needs of the society.	22	6.0 (5.0 to 7.0)
8. If lay public disagrees with the research topic, then the topic should be changed.	23	5.0 (4.0 to 6.0)
9. When planning a research project, researchers should involve the public in order to determine the needs of the society related to the project.	22	6.0 (6.0 to 7.0)
10. Presence of different stakeholders in the research process would only disturb the researchers.‡	21	3.0 (2.6 to 4.0)

RRI – responsible research and innovation, Md – median, CI – confidence interval.

*Number of respondents for each question, as some answers were not provided by the participants.

†Numbers refer to selected scores on Likert answer scale with 7 scoring points, from 1(“strongly disagree”) to 7 (“strongly agree”), with 4 as a neutral point (“neither agree nor disagree”).

‡Negative statements, reverse scoring was used.

Museum trainers’ comments showed that they agreed that the programs were motivating and highly interactive, and that the resources were appropriate. They also expressed intention for future use and promotion of the training among their colleagues. Commonly addressed topics in the open-ended answers are presented in Table 10, and those indicate that participants perceived museum activities as motivating and interesting, but also required more time and more examples. A sample of open-ended answers is available in Table s12 in the Supplement.

## Discussion

Overall, satisfaction with HEIRRI training programs on responsible research and innovation was high, both for participants and for the trainers. Intention to change behaviour in the future using RRI principles was higher in non-consortium participants, as well as their attitudes towards RRI. Participants’ comments were brief and positive, and there was no difference in linguistic characteristics of the feedback by gender, consortium status, or previous research activities. More time and flexibility, as well as cases from a variety of disciplines, were the main suggestions for improvement of the programs.

### Strengths and limitations

As the HEIRRI project tested pilot training programs, our main focus was to collect feedback on their major strengths and weaknesses. The pilot provided us with a comprehensive quantitative and qualitative insight that informed recommendations for improvement of the final programs [22]. One major limitation of this research is the large dropout of participants for evaluation surveys. However, small response rate is expected for online surveys [23]. Future assessments might use reminders and push notifications to improve response rate [23]. Another limitation is that the survey items did not include research area or discipline of the participants. In the future, these training programs could be followed up by more detailed measurements, with a strong focus on RRI outcomes.

### Interpretation

Our study showed that HEIRRI training programs are feasible and interactive forms of RRI education, successfully implemented in different settings and by different trainers. They constitute a toolbox of engaging activities that can be flexibly used for introduction, development, and longitudinal involvement of higher education institutions in RRI values. Our pilot results showed that these programs offered a participatory space for students to reflect and share opinions. The state-of-the-art review on RRI education [13] recognized the need for teaching within the real societal and professional context, which can be accomplished through using real life dilemmas, cases and practical approaches. HEIRRI programs are based on these principles, with the aim to engage participants both emotionally and intellectually, and evaluation results indicate that these aims were reached.

RRI as a concept does not have a long history in higher education institutions, even though some of the key dimensions of RRI are inevitably a part of existing education, such as research ethics or open access. However, several European Union projects relate to RRI teaching [13]. Some of them focus on specific relationships of RRI, with open science [24], or with institutions and grounded actions for integration with society [25], as well as promotion of RRI integration across academia, business, and civil society organizations [26]. The project EnRRICH (Enhancing Responsible Research and Innovation through Curricula in Higher Education) aimed to improve capacity of higher education institutions’ staff and students for knowledge, skills and attitudes to support RRI embedding in curricula [27]. They created a range of case-based debates and Science Shops, in which they closely collaborated with civil society organizations in order to recognize problems and important questions. These questions were then rephrased into research projects, which were conducted by the Science Shop researchers and students [28]. Their aim was to help academics realize that RRI concepts already exist in their work and are not a new requirement [29]. HEIRRI aimed to create RRI educational tools that will help integrate RRI through different levels of education, including practicing researchers, but also students, high school, undergraduate, and graduate, who will then learn to think, anticipate and observe their future careers through the RRI prism [14]. Both HEIRRI and EnRRICH emphasized the importance of phronesis, practical knowledge and wisdom, in determining the best course of action in uncertain and controversial situations [30, 31].

Since the HEIRRI project was completed, a number of RRI educational interventions were developed. Most of them used the same educational tools as HEIRRI: case oriented [32], dialogue centered [33], inquiry-based learning [34], with public engagement [35]. Some interventions for encouraging anticipatory thinking, transparency, inclusion and reflection, all of which are important parts of RRI, are based on body postures, which are supposed to orient the body and the mind towards humility and openness [36]. Great emphasis is put on the relationship of power and posture. While HEIRRI does not directly address postures in its RRI framework, it does indirectly, by encouraging a participatory discussion and removing the power role of the teacher at the front of the classroom. In some of the activities, the teacher plays a passive role, and in the others the teacher moderates the discussion, suppressing the use of technical terms and ensuring equal participation in the conversation [15].

These interventions were evaluated using different methods, either interviews, pre and post-test surveys, or both, but they were mostly performed on small sample sizes and varied in outcomes [33, 37]. This reflects the complexity of RRI values and ways in which they should be measured. A set of RRI key indicators in education has been developed to help evaluate RRI outcomes [38], which were applied in a qualitative study focused on public-science engagement [39]. They helped establish the importance of direct engagement on motivation for research for societal benefit [39]. However, there is still a need for a comprehensive evaluation of RRI education in other RRI dimensions.

Training instructions that allowed trainers’ freedom and highly adaptable activities were the main strengths of HEIRRI programs. For successful implementation, motivated and committed trainers were recognized as key enablers and drivers of institutional changes, which the European Commission describes as interventions that have meaningful impact in the RRI dimensions [40]. These changes include, but are not limited to, ethics and integrity training, training on implicit bias, citizen science involvement, research results communication training, and implementation of an RRI policy development action plan [40]. RRI educational activities could be a first step towards these initiatives. They are already a part of a 36-item list of RRI indicators developed by the MoRRI (Monitoring the evolution and benefits of Responsible Research and Innovation) project [41], which are recommended to use for monitoring of science and innovation systems [42].

One of the concerns participants raised about the HEIRRI programs was underrepresentation of the humanities in case examples and scenarios. RRI has historically been linked to science and technology, and adaptations and nuances of RRI activities are still more developed in natural sciences [43]. Previous research has shown that RRI can be used as a mending tool for unsatisfactory interdisciplinary collaboration between social sciences and humanities and natural sciences [32]. In the final version of HEIRRI programs, we addressed participants’ comments and included more examples from the field of humanities [15]. These educational activities allow for a high level of flexibility. It is expected that they will be adapted to specific institutional needs, and that cases and examples will be modified for specific contexts.

Non-consortium organization participants, from India, Algeria, Bulgaria, Lithuania, Bosnia and Herzegovina, Germany, and Spain, had overall more positive attitudes towards RRI, as well as higher intentions to use RRI principles in the future. It is likely that participants from these institutions were first introduced to RRI through HEIRRI training programs, while consortium institutions participants were already familiar with the concept. Results imply that non-consortium trainings were inspirational and exciting in greater measure than consortium trainings. This might make HEIRRI programs particularly suitable for introducing RRI in new educational settings to participants who have no previous RRI experience or knowledge.

The Framework for Qualifications of the European Higher Education Area states that students finishing the master’s level have to “have the ability to integrate knowledge and handle complexity, and formulate judgements with incomplete or limited information, but that include reflecting on social and ethical responsibilities linked to the application of their knowledge and judgements” [44]. Young researchers should be able to understand the importance and accountability that accompanies their work. They need to be able to see the entirety of inclusive societal and environmental sciences and find their respective place in their environment. In order to produce generations of not only skilled, but also conscious and socially aware individuals, higher education institutions need to foster their development from the beginning. To achieve this, RRI key dimensions should be integrated in their education. In the world that has known COVID-19 and societal equity movements, it is vital that societal responsibility is cultivated, assessed, and continually advanced [36].

### Recommendations

Participants’ feedback from the surveys and open-ended questions were used to identify areas that need improvement and shape recommendations for final versions of the HEIRRI programs. These recommendations were organized in three categories: organizational, implementation, and materials and resources. For organizational issues, the focus was on the inclusion of the programs in existing curricula. During the piloting of HEIRRI courses we paid special attention to trainers’ previous experience in RRI teaching, and we found that the inclusion of the programs in higher education institutions was not a major issue. However, trainer’s previous experience in RRI was shown to be important. We recommend that users of the HEIRRI programs consider the importance of having motivated and committed trainers, as they are the key organizational enablers.

For implementation issues, we focused on timing, including time needed for preparation, timing of activities, and balance of the workload and credits participants could receive. HEIRRI programs have already been designed to allow for a high level of flexibility. Their design considered adaptation for specific needs and contexts. In addition, we recommend that users consider segmenting the training activities into two- to four-hour blocks, spread over a couple of days or weeks.

Although we added materials relating specifically to humanities to HEIRRI programs, we recommend that trainers consider their local context when preparing the courses. If possible, they should consider disciplines and contexts of their participants and take those into account.

Finally, we emphasize and promote trainer freedom and flexibility in adapting the training materials.

## Conclusion

As a discipline that transforms and guides the development of society, science has remained secluded and elusive to its actors and the public for far too long. RRI shifts science from a closed and autonomic discipline to a democratic one [45]. In order to successfully transfer science to a community and become an inclusive, transparent and open practice, one of the goals of higher education institutions and researchers should be raising awareness and enabling public and young researchers with skills to recognize and apply RRI values. HEIRRI training is suitable for a range of different disciplines, including forensic science, and is free to use and adapt to specific educational and research circumstances (available from: https://rri-tools.eu/heirri-training-programmes) (15). For any discipline that dwells into human behaviour and complex relationships with other humans and their environment, both current and future, RRI provides a lens through which we can recognize what needs to change and how to accomplish those changes in a sustainable and responsible way. As a toolbox of activities that are flexible and engaging, HEIRRI training can be used to introduce RRI at all levels of higher education and to members of the public interested in research and innovation processes.

## Author contributions

RT, IB, NM, MC, AL, GR, AM and the HEIRRI Consortium conceived the study, participated in its design and coordination. RT drafted the manuscript; IB performed the statistical analysis. All authors contributed to the final manuscript and approved it.

## Supplementary Material

Supplemental Material
