# Supplementary material for: Responsible research and innovation training programs: implementation and evaluation of the HEIRRI project
Source: Forensic Sci Res. 2021 Nov 2;6(4):320–30. doi: 10.1080/20961790.2021.1970319 (PMC8815623; doi:10.1080/20961790.2021.1970319)
Supplement: Supplemental Material [file TFSR_A_1970319_SM8065.docx]

**Supplement**

**Table s1.** Overview of HEIRRI training programs (adapted from Lang [14, p.23])

| **#** | **Name** | **Audience** | **Design** |
| --- | --- | --- | --- |
| 1 | Studying Responsibility: A Module-Based Integration of RRI into Bachelor Programmes | Bachelor students | This training programme for bachelor’s students is composed of four modules, which can either be implemented together or incorporated in existing courses. In the adaptable modules, students: 1) learn what research, innovation. and RRI mean in their field of study; 2) deal with concrete cases of RRI; (3) learn practical approaches to promote RRI; and 4) learn to reflect on the responsibility of R&I in their scientific field. |
| 2 | Doing and Experiencing Dialogical Reflection on Research and Innovation | Master students | In this interactive course students get to know and discuss different approaches of how to facilitate dialogues on issues of R&I. They then develop dialogue activities in groups and implement these in “dialogue experiments” with their colleagues as participants. |
| 3 | Enhance your Thesis | Master students | In this course for master’s students starting their final thesis, participants get to know concepts of RRI and discuss case examples, and then identify, investigate, and reflect on RRI aspects of their own theses. |
| 4 | Responsible PhD: RRI and PhD Research Projects | PhD students | This seminar introduces PhD students to the concept of RRI, discussing its role in (academic) research by case examples, and shows how PhD students can apply RRI in their own research. |
| 5 | Supporting RRI: Developing RRI Guidelines for PhD Candidates | PhD students | In this five- to six-hour workshop for PhD candidates, students develop RRI guidelines specific for the position of a PhD researcher. They identify their own possibilities to promote change towards more responsible research within their research context, and then set up guidelines that consider different concepts of RRI. |
| 6 | Teaching Responsible Research and Innovation in Higher Education | Academic and non-academic higher education institution members | This train-the-trainer online course is based on the participants’ independent study of provided texts, video material, and literature, in combination with writing assignments and discussions in an online forum and chat. Participants deal in three parts with: 1) concepts and the relevance of RRI; 2) practical approaches towards RRI; and (3) teaching RRI in higher education. |
| 7 | Facilitating Reflection on Responsible Research and Innovation | Academic and non-academic higher education institution members | In this train-the-trainer one-day workshop participants experience a reflection exercise on issues of RRI and related aspects. They learn how to initiate and facilitate such reflection in their own field and particularly in teaching. |
| 8 | Considering Responsible Research and Innovation by Design | Master’s and PhD students, R&I actors and other stakeholders | This interactive five-day summer school brings together participants with different scientific and professional backgrounds to learn about RRI and work on concrete research proposals that consider RRI in organising a research process. |
| 9 | Concepts and Practice of Responsible Research and Innovation | Students, researchers, higher education institutions actors, stakeholders, and other interested actors | This massive open online course (MOOC) is directed at a broader audience, from students and other stakeholders and actors of higher education institutions (e.g. researcher, librarians, administrative staff) to other interested people. An overview of existing concepts and practices of responsible and sustainable research and development is given. |
| 10 | Science Open to Society. Schools Open to Science. | Secondary school teachers in training | This training programme supports future secondary school teachers to deal with and discuss issues of responsibility with regard to science, research, and innovation with their own students. |

**Table s2. Piloted training programs in HEIRRI consortium**

| **Higher education institution** | **Country** | **Programme** | **Date** |
| --- | --- | --- | --- |
| Universitat Pompeu Fabra (UPF) | Spain | #08 Summer school* | 12-15 Sep 2017 |
|  |  | #02 Master’s | Oct 2017 |
|  |  | #07 Train the trainer | 21 Nov 2017 |
|  |  | #01 Bachelor’s | 2 Oct 2017, 30 Jan, 6, 16 Feb, 21-22 Feb, 1-2 Mar, 6-7 Mar 2018 |
|  |  | #10 Secondary school teachers | 23, 29 Jan, 1 Feb 2018 |
|  |  | #09 MOOC | 26 Feb – 15 Apr 2018 |
| University of Split | Croatia | #01 Bachelor’s | 4, 19 Oct 2017 |
|  |  | #08 Summer school | 16-20 Oct 2017 |
|  |  | #04 PhD | 24 Nov 2017 |
|  |  | #07 Train the trainer | 1 Feb 2018 |
| Aarhus University | Denmark | #04 PhD† | 29-30 Nov 2017 |
|  |  | #05 PhD | 23 Apr 2018 |
| University of Bergen | Norway | #03 Master’s | Aug-Sep 2017 |
|  |  | #04 PhD | 5 Apr 2018 |
|  |  | #05 PhD | 4 Apr 2018 |
| Institute for Advanced Studies | Austria | #01 Bachelor’s‡ | Oct 2017 - Jan 2018 |
|  |  | #07 Train the trainer‡ | 20 Apr 2018 |

*incl. CosmoCaixa museum;

†incl. Steno science museum; ^±^ in collaboration with the Lauder Business School (LBS), Vienna, Austria

**Table s3. Piloted training programs outside the HEIRRI consortium**

| **Higher education institution** | **Country** | **Programme** | **Date** |
| --- | --- | --- | --- |
| Jawaharlal Nehru University | India | 8 | 30 Jan 2018 |
| University of Calcutta |  | 7 | 20 Jan 2018 |
| Pan African University Institute of Water & Energy Sciences | Algeria | 7 | 16 Dec 2017 |
|  |  | 8 | 17-21 Dec 2017 |
| Christian-Albrechts Universität zu Kiel | Germany | 4 | 17 Jan 2018 |
|  |  | 7 | 9 Feb 2018 |
| Universitat Autònoma de Barcelona (UAB) | Spain | 4 | 12 Feb 2018 |
|  |  | 4 | 26 Feb 2018 |
|  |  | 7 | 13 Feb 2018 |
|  |  | 7 | 27 Feb 2018 |
| Aachen University | Germany | 4 | 21 Feb 2018 |
|  |  | 7 | 7 Mar 2018 |
| Universitat Jaume I | Spain | 4 | 23 Nov 2017 |
|  |  | 4 | 14 Dec 2017 |
| Sofia University | Bulgaria | 4 | 4 Dec 2017 |
|  |  | 7 | 16 Dec 2017 |
| University of Mostar School of Medicine | Bosnia and Herzegovina | 4 | 24 Nov 2017 |
|  |  | 7 | 16 Mar 2018 |
| Mykolas Romeris University | Lithuania | 4 | 18 Nov 2017 |
|  |  | 7 | 16 Nov 2017 |

Survey items for training participants:

**Table s4.** Course assessment survey items (*level 1* outcomes)

| Items |
| --- |
| 1. This is a good way for learning responsible research and innovation RRI. |
| 2. More real-world examples should be presented during the course. |
| 3. The course covered too much content in a short period of time.* |
| 4. The practical work we have to do during the course was very helpful in understanding the topic. |
| 5. The resources available during the course covered the course content very well. |
| 6. This course helped me to better understand the importance of responsible research and innovation RRI in research. |
| 7. The course had a very practical and hands on approach. |
| 8. I could not relate the course content to training for my profession degree.* |
| 9. The course topics were related to my study interests or research work. |
| 10. The course did not help me to understand the importance of responsible research and innovation (RRI).* |
| 11. The course materials were presented in a clear and logical way. |

*Reversely scored

**Table s5.** Attitude towards RRI survey items (*level 2a* outcomes)

| Items |
| --- |
| 1. It is not necessary to include all individuals or groups with interest and concern in the topic of the research (stakeholders) in the research process.* |
| 2. The inclusion of many diverse individuals or groups with interest and concern in the topic of the research (stakeholders) may cause confusion in the research process.* |
| 3. Inclusion of the public in research could be an obstacle to the research process itself.* |
| 4. When developing a new research project, individuals or groups with interest and concern in the topic of the research (stakeholders) should be included already at the planning stage. |
| 5. Before starting a new research project there should be an open discussion to address the needs of the society. |
| 6. Involving individuals or groups with interest and concern in the topic of the research (stakeholders) in the research process reduces the autonomy of a researcher.* |
| 7. Societal values do not have a significant impact on the research process.* |
| 8. The whole research process should be transparent and publicly available from the start of the project. |
| 9. Researchers should change the aims of their research if the public demands it. |
| 10. The whole research process is actually a dialogue between the researcher and the society. |
| 11. There should be ways to store research data and make them publicly available for a longer period. |
| 12. Researchers should discuss the usefulness of research findings even after the research has finished. |
| 13. Researchers should publish all the results of their studies and not only some of them. |
| 14. Researchers should serve the society. |

*Reversely scored

**Table s6.** Behavioral intentions survey items (*level 3a* outcomes)

| RRI dimension |  |
| --- | --- |
| Anticipation | In future I will be open to act on future challenges keeping in mind how my work or research shapes the future. |
| Reflection | In my future work or research, I will think more carefully about the problems at issue and practices to deal with them, including my own and my institution’s values and rules. |
| Openness and Transparency | In future, I will share the information on my research and its results in a way that makes sense to different audiences that may be interested in my work or research. |
| Responsiveness | In future I will take into account new knowledge perspectives views and rules when planning and doing my work or research. |
| Adaptation to change | In future I will adapt to new circumstances and insights as well as the values of all those concerned by my work or research. |

**Table s7.** Course satisfaction for student participants

| Items | *n** | Response (Md, 95% CI)† |
| --- | --- | --- |
| 1. This is a good way for learning responsible research and innovation RRI. | 507 | 6.0 (6.0 to 6.0) |
| 2. More real-world examples should be presented during the course. | 507 | 5.0 (4.0 to 5.0) |
| 3. The course covered too much content in a short period of time.‡ | 500 | 4.0 (4.0 to 4.0) |
| 4. The practical work we have to do during the course was very helpful in understanding the topic. | 505 | 6.0 (6.0 to 6.0) |
| 5. The resources available during the course covered the course content very well. | 503 | 6.0 (6.0 to 6.0) |
| 6. This course helped me to better understand the importance of responsible research and innovation RRI in research. | 505 | 6.0 (6.0 to 6.0) |
| 7. The course had a very practical and hands on approach. | 500 | 6.0 (6.0 to 6.0) |
| 8. I could not relate the course content to training for my profession degree.‡ | 502 | 2.0 (2.0 to 2.0) |
| 9. The course topics were related to my study interests or research work. | 500 | 6.0 (6.0 to 6.0) |
| 10. The course did not help me to understand the importance of responsible research and innovation RRI .‡ | 500 | 1.0 (1.0 to 2.0) |
| 11. The course materials were presented in a clear and logical way. | 500 | 6.0 (6.0 to 6.0) |

Md – median, CI – confidence interval

* *n* = number of respondents for each question, as some answers were not provided by the participants.

†Numbers refer to selected scores on Likert answer scale with 7 scoring points, from 1 – “strongly disagree” to 7 – “strongly agree”, with 4 as a neutral point (“neither agree nor disagree”).

‡Negative statements, reverse scoring was used and presented here.

**Table s8.** Attitudes towards responsible research and innovation (RRI) for student participants

| Items | *n** | Response (Md, 95% CI)† |
| --- | --- | --- |
| 1. It is not necessary to include all individuals or groups with interest and concern in the topic of the research (stakeholders) in the research process.‡ | 486 | 2.0 (2.0 to 3.0) |
| 2. The inclusion of many diverse individuals or groups with interest and concern in the topic of the research (stakeholders) may cause confusion in the research process.‡ | 488 | 4.0 (4.0 to 4.0) |
| 3. Inclusion of the public in research could be an obstacle to the research process itself.‡ | 485 | 3.0 (3.0 to 4.0) |
| 4. When developing a new research project, individuals or groups with interest and concern in the topic of the research (stakeholders) should be included already at the planning stage. | 487 | 6.0 (6.0-6.0) |
| 5. Before starting a new research project there should be an open discussion to address the needs of the society. | 485 | 6.0 (6.0-6.0) |
| 6. Involving individuals or groups with interest and concern in the topic of the research (stakeholders) in the research process reduces the autonomy of a researcher.‡ | 483 | 4.0 (4.0 to 4.0) |
| 7. Societal values do not have a significant impact on the research process.‡ | 478 | 2.0 (2.0 to 2.0) |
| 8. The whole research process should be transparent and publicly available from the start of the project. | 485 | 6.0 (6.0-6.0) |
| 9. Researchers should change the aims of their research if the public demands it. | 479 | 4.0 (4.0 to 4.0) |
| 10. The whole research process is actually a dialogue between the researcher and the society. | 476 | 5.0 (5.0 to 5.0) |
| 11. There should be ways to store research data and make them publicly available for a longer period. | 477 | 7.0 (6.0 to 7.0) |
| 12. Researchers should discuss the usefulness of research findings even after the research has finished. | 476 | 7.0 (6.0 to 7.0) |
| 13. Researchers should publish all the results of their studies and not only some of them. | 479 | 7.0 (6.0 to 7.0) |
| 14. Researchers should serve the society. | 477 | 6.0 (6.0 to 7.0) |

Md- median, CI- confidence interval.

*N = number of respondents for each question, as some answers were not provided by the participants.

†Numbers refer to selected scores on Likert answer scale with 7 scoring points, from 1 (“strongly disagree”) to 7 (“strongly agree”), with 4 as a neutral point (“neither agree nor disagree”).

‡Negative statements, reverse scoring was used.

**Table s9.** Attitudes towards RRI dimensions for student participants

| RRI dimension |  | *n** | Response (Md, 95% CI)† |
| --- | --- | --- | --- |
| Anticipation | In future I will be opened [sic] to act on future challenges keeping in mind how my work or research shapes the future. | 474 | 7.0 (6.0 to 7.0) |
| Reflection | In my future work or research, I will think more carefully about the problems at issue and practices to deal with them, including my own and my institution’s values and rules. | 478 | 7.0 (6.0 to 7.0) |
| Openness and Transparency | In future, I will share the information on my research and its results in a way that makes sense to different audiences that may be interested in my work or research. | 475 | 7.0 (6.0 to 7.0) |
| Responsiveness | In future I will take into account new knowledge perspectives views and rules when planning and doing my work or research. | 472 | 7.0 (7.0 to 7.0) |
| Adaptation to change | In future I will adapt to new circumstances and insights as well as the values of all those concerned by my work or research. | 452 | 7.0 (6.0 to 7.0) |

Md-median, CI- confidence interval

* *n* = number of respondents for each question, as some answers were not provided by the participants.

†Data presented as median (Md) response and 95% confidence interval (CI). Numbers refer to selected scores on Likert answer scale with 7 scoring points, from 1 – “strongly disagree” to 7 – “strongly agree”, with 4 as a neutral point (“neither agree nor disagree”)

**Table s10.** Open-ended question and selected answers from student participants in training pilots

| **Please give us the feedback on the course in your own words:** |
| --- |
| Very good intro movie. Nice overview on the scope of RRI. More concrete examples/material for training purposes in home institution would have been helpful. |
| I liked the fact that the learning process was done collectively by sharing opinions, questions, comments, and assignments in the forum. This resonates a lot with the spirit that RRI wants to put forward. However, I also think that the course was way too short! |
| In general lines I am very satisfied with the course. What I liked most about the course It has been to be able to learn more about the RRI framework, its dimensions and its social agendas. I would also like to point out that the course materials have served as a perfect introduction to this framework and the discussion among students has been very interesting and productive. Perhaps, due to the very nature of the RRI framework, the materials have to be continually reviewed and updated each course. |
| Thank you for this course! |
| The contents of the course were really comprehensive and well-explained. However better explanations of the given tasks would have helped in some cases. |
| It is very helpful to listen to the ideas and opinions of fellow PhD students from different fields. I think it was a good opportunity to self-reflect and grasp the main concepts and challenges of RRI. |
| Fun, good, learned a lot, good discussions |
| Practical and hands-on approach is the way. Good job! |
| I think the course was relevant to the future of research in Africa. It was time constrained and also a lack of more examples that expound on responsible research and innovation. Otherwise it was a good course and crucial to future researches [sic]. |
| Maybe add more real examples about how to incorporate RRI in specific projects. |
| I would increase the number of hours for the course to understand better the concepts. |
| This course is excellent. Really stimulates the thinking about the research work and work in general. It helped me to find the answers on some personal dilemmas and I've learned a lot on principles in research and publishing work. It certainly made an impression, and I am happy that I was a part of it. |
| I found the course very structured and well planned. I also appreciated how the people holding the lectures/seminars were energetic and talkative; making the students take part and discuss. |
| I think this course is very dynamic, interactive, interesting and useful. Thank you! |
| This course, in general, seems to me like a very useful tool for researchers from all disciplines. It breaks the stereotype of the isolated researcher aiming for pure knowledge. (…) It has been a dynamic and interesting exercise that, from my point of view, has raised awareness to all of us on applying more social, accessible and responsible processes in our different research fields. |

**Table s11.** Open-ended question and selected answers from pilot trainers

| **Please give us the feedback on the course in your own words – what was good, what needs improvement and how you can engage more in RRI training:** |
| --- |
| I taught the one-day workshop for PhD students - it was a great experience. The only wish we (the teacher and the students) had was that we would have liked to have more time to cover all important aspects of RRI. The students were motivated and very eager to implement RRI in their research. |
| (…) The RRI topics were integrated in the course on research methodology in biomedicine and health, and it worked really well. It was easy to implement the pilot and it fit nicely with the topics that we teach and students were really very impressed with the topic and the need to integrate it their curriculum. I would call this a great success, which resulted in our decision to introduce RRI into courses for all undergraduate/graduate programs (medicine, dental medicine, pharmacy, and health studies). Well done, HEIRRI! |
| The course was very good but not sure we provided the "how to do it" but we most focused on the concept. More time would allow to go further. |
| (…) All in all, the pilot designs #4 and #5 are OK but a little over-programmed, (…). This is OK as long as teachers pick and choose themselves, so also because of this it is a good idea to emphasize the need for contextualisation and adaptation. |
| (…) Participants are providing very good feedback about the materials and activities. They value the flexibility of an on-line course that this, as well as the opportunities for sharing ideas and collective reflection through the on-line Forum. In next editions, we will include more on-line spaces for discussion, as suggested by participants, and try to make the course longer. |
| Methodologies proposed were original and stimulating. Some of the timing proposed were unrealistic to carry on groups with many participants. |

**Table s12.** Open-ended questions and selected answers from participants in museum activities

| **Do you have suggestions for the improvement of for future activities related to responsible research and innovation?** |
| --- |
| This activity is very dynamic and in my opinion is good. |
| It was interesting to hear about other opinions. |
| Although the audience is mostly women (30 of 43), most of the interventions have been from men. Participation from women should be more promoted. |
| **Did this activity made you think about your previous opinions and ideas about the research presented? Will it change your opinions or practices?** |
| Possibly. The inter-generational collaboration is important. |
| No. I think it has been a very interesting debate, but the topic of research has become a bit "diluted". |
| It has made me realise how little informed I am, and about the importance of thinking and working with scientific rigour. |
| Yes, and yes. |
| Yes. |
| **Did all relevant representatives from the society participate in this activity?** |
| Yes, there was a mix of people from different associations. |
| Yes, there was a lot of people and very diverse. |
| Yes, diversity helps connect better with social demands |
| Yes. Profiles were very diverse, including the end-users. |
| No, people with no education should have been included. |
| **Do you think responsible research is important?** |
| Yes, it is not only important, it is fundamental. It is a pity that in this country the necessary resources are not destined to it. |
| Yes, because research has to respond to clear needs with a limited budget. The level of involvement of researchers in societal needs is vital. |
| Yes. It is important to guarantee the proper use of resources and promote society's trust in science. |
| Yes, because it affects us all. |
| Yes, because we have a direct involvement in the present and future! |
| **Do you have any other comments?** |
| It would be great to organize more debates like this, it was very interesting. |
| Nice project. |
| Thank you. |
